# CDK4/6 Inhibitors in Breast Cancer—Who Should Receive Them?

**DOI:** 10.3390/ijms262110322

**Published:** 2025-10-23

**Authors:** Anran Chen, Ze-Yi Zheng, Meenakshi Anurag, Ahmed Elkhanany, Natalie C. Chen, Eric C. Chang

**Affiliations:** 1 Research Oncology, Bayer, Cambridge, MA 02142, USA; anran.chen@hotmail.com; 2 Lester and Sue Smith Breast Center, Baylor College of Medicine, Houston, TX 77030, USA; zeyiz@bcm.edu (Z.-Y.Z.); meenakshi.anurag@bcm.edu (M.A.); ahmed.elkhanany@bcm.edu (A.E.); natalie.chen@bcm.edu (N.C.C.); 3 Department of Medicine, Baylor College of Medicine, Houston, TX 77030, USA; 4 Department of Molecular and Cellular Biology, Baylor College of Medicine, Houston, TX 77030, USA

**Keywords:** RAS, RAF, estrogen receptor, CDK4/6, breast cancer, neurofibromatosis

## Abstract

More than 70% of breast cancers are estrogen receptor-positive (ER^+^). Endocrine therapy that blocks estrogen signaling remains the cornerstone of treatment, yet relapses continue to affect many patients. Cyclin-dependent kinases 4 and 6 (CDK4/6) regulate the G1-S phase transition in the cell cycle, and pharmacological inhibition of this pathway has been successfully leveraged to reduce recurrence. CDK4/6 inhibitors combined with endocrine therapy are now the standard of care, although determining the optimal patient population for treatment remains a key challenge. A newly published study provides important insight, showing that loss of the NF1/neurofibromin tumor suppressor confers greater sensitivity to CDK4/6 inhibition, as these tumors rely heavily on CDK4/6 activity for survival under endocrine therapy.

Cyclin-dependent kinases 4 and 6 (CDK4/6), in complex with D-type cyclins, are critical regulators of the G_1_-S phase transition of cell cycle [1,2]. In the resting state, the active, hypophosphorylated retinoblastoma (Rb) tumor suppressor binds and inactivates the E2F transcription factor, thereby blocking cell cycle progression. Following mitogenic stimulation, cyclin D levels rise and activate CDK4/6, which in turn phosphorylate Rb. This modification disables Rb’s inhibitory function, releasing E2F to drive transcription of genes required for DNA synthesis and irreversibly committing the cell to division. The importance of this pathway in cancer is underscored by its frequent subversion through cyclin D1 overexpression or loss of inhibitors such as p16. Although this CDK4/6-Rb pathway is frequently altered in cancers, it is not strictly essential for cell division, as deletion of *Ccnd1*, *Cdk4*, or *Cdk6* is not embryonically lethal in mice. These observations support the concept that CDK4/6 represent prime targets for anticancer therapies with limited toxic to normal cells [1,2].

Approximately 70–80% of breast cancers express estrogen receptor α (ER), a ligand-dependent transcription factor [3]. In the presence of estrogen, ER associates with co-activators to induce expression of growth-promoting genes, including *CCND1*, which encodes cyclin D1. ER^+^ breast cancers are treated with endocrine therapies that disrupt estrogen signaling. For example, tamoxifen is a selective ER modulator (SERM) that antagonizes ER signaling in the breast by recruiting co-repressors to chromatin [4]. Additional treatment options include fulvestrant, a selective ER degrader (SERD), and aromatase inhibitors, such as letrozole and anastrozole, which block estrogen synthesis in postmenopausal women. Despite advances in targeting ER with tamoxifen and aromatase inhibitors, outcomes for ER^+^ disease have not improved as rapidly as for ER^−^ or human epidermal growth factor receptor 2 positive (HER2^+^) breast cancer [5]. Moreover, ER^+^ tumors account for the majority of global breast cancer deaths, now exceeding half a million annually [6].

Over recent years, several CDK4/6 inhibitors (CDK4/6i), such as palbociclib, abemaciclib, and ribociclib, have been developed for the treatment of ER^+^ breast cancer, achieving significant clinical success. ER-positivity is currently the primary biomarker guiding CDK4/6i therapy, as ER^+^ breast cancer cell lines demonstrate the strongest response in preclinical studies [7]. Conversely, ER-negative cell lines generally show little response, partly because they lack functional Rb, underscoring the importance of Rb status in determining CDK4/6i response (see below).

In first-line metastatic settings [8], the PALOMA-2 (palbociclib, NCT01740427), MONALEESA-2 and -7 (ribociclib, NCT01958021 and NCT02278120, respectively), and MONARCH-3 (abemaciclib, NCT02246621) trials demonstrated that adding a CDK4/6i to endocrine therapy improved progression free survival (PFS) in ER^+^/HER2^−^ metastatic breast cancer by approximately 50%. Similar benefits were observed in the second-line PALOMA-3 (NCT01942135), MONALEESA-3 (NCT02422615) and MONARCH-2 (NCT02107703) trials. Notably, both ribociclib and abemaciclib have also demonstrated overall survival (OS) benefits in the metastatic setting. In early-stage ER^+^/HER2^−^ disease, two years of adjuvant abemaciclib and three years of ribociclib modestly reduce recurrence rates in high-risk disease; however, neither agent has yet shown an OS advantage [8]. Health economic analyses indicate that CDK4/6i plus letrozole is not cost effective in the first-line metastatic setting [9], and concerns about toxicity and quality of life can also limit their use in the adjuvant context [10]. Together, these observations raise a fundamental question: are all ER^+^ breast cancers intrinsically resistant to CDK4/6 inhibition or does a subset of patients derive the greatest benefit?

Because CDK4/6 promote cell cycle progression through Rb inactivation, loss of functional Rb could identify patients intrinsically resistant to CDK4/6i therapy [11]. Indeed, *RB1* mutations frequently emerge after CDK4/6i treatment, and their role in promoting resistance has been experimentally validated [12,13]. However, *RB1* mutations are rare in untreated population (<1%) [12,14], and most ER^+^ tumors retain Rb expression (>90%), as determined by immunohistochemistry (IHC) [15]. This suggests that Rb has essential functions in ER^+^ breast cancer, consistent with the observation that *Rb1*-null mice are embryonically lethal. In addition, high level of cyclin E1 expression, which activates CDK2, has also been associated with resistance to CDK4/6i in PALOMA-3 study [16]. Other acquired resistance mechanisms include activation of RAS, AKT1, and AURKA [13].

Among patients whose tumors are resistant to endocrine therapy, CDK4/6i treatment represents a top priority to reduce recurrence. *ESR1* mutations in the ligand-binding domain drive resistance to aromatase inhibitors; however, in the PALOMA-3 study, adding palbociclib to fulvestrant significantly improves PFS even in patients with baseline *ESR1* mutations [17]. In the same vein, *ESR1*-fusions represent another class of aberrant ER with enhanced signaling capacity, and preclinical models carrying these features also respond to CDK4/6i [18]. Furthermore, a subset of ER^+^ breast cancers with defects in MutL mismatch repair [19] and DNA damage repair [20] exhibit resistance to all classes of endocrine therapy, yet preclinical data indicate that such tumors still respond to CDK4/6i.

Taking a precision medicine perspective, a recent study by Zheng et al. published in *Science Translational Medicine* demonstrated that a subset of ER^+^ breast cancers exhibit heightened sensitivity to CDK4/6i, owing to their pronounced reliance on CDK4/6 activity for survival in the context of endocrine therapy [21]. This study centers on the neurofibromin/NF1 tumor suppressor, which functions both as a GTPase activating protein (GAP) that represses RAS (rat sarcoma virus) signaling and as a canonical ER transcriptional co-repressor that inhibits ER signaling [22]. NF1 inactivation therefore leads to simultaneous activation of RAS and ER signaling (Figure 1). Consistent with its co-repressor activity, loss of NF1 enhances recruitment of ER to the chromatin, including at the *CCND1* locus, resulting in increased cyclin D1 levels and Rb phosphorylation. To demonstrate the clinical relevance of this mechanism, the authors show that *NF1* mRNA levels inversely correlate with cyclin D1 protein levels in TCGA’s Reverse-Phase Protein Array (RPPA) dataset. Furthermore, in two separate early-stage ER^+^/HER2^−^ cohorts, tumors harboring both an *NF1* shallow deletion and high *CCND1* expression have worse relapse-free survival (RFS) than those with either abnormality alone.

While cyclin D1 is required for CDK4/6 activation, full activation of protein kinases also depends on phosphorylation of a threonine residue within the activation loop, which permits substrate access to the catalytic site. CDK4/6 are structurally distinct from other cyclin-dependent kinases in the activation loop [23]. For example, CDK2 is phosphorylated at T160, with an adjacent histidine at position 162. In contrast, the corresponding threonine in CDK4 is T172, which is followed by a proline rather than a histidine at position 173. Because proline imposes strong conformational constraints, this substitution can significantly alter the local structure of the activation loop and thereby influence binding of the CDK-activating kinase (CAK). The identity of the cognate CAK that phosphorylates CDK4 at T172 in ER^+^ breast cancer cells remains elusive. Although CDK7 is a known CAK for CDK2 and can phosphorylate CDK4 in vitro, it requires mM concentrations of ATP [24]. Speculating that CAK substrates possess distinct amino acid sequences in their activation loops, Zheng et al. performed an unbiased Group-based Prediction System analysis [25] on the CDK4 sequence, which identified both CDK7 and CRAF/RAF1 (rapidly accelerated fibrosarcoma 1) as potential CAKs. The latter is particularly notable given that RAF activity is stimulated upon NF1 loss [21]. Indeed, in vitro kinase assays using purified components and 10 µM ATP showed that CRAF efficiently phosphorylates purified CDK4 at T172, whereas CDK7 and c-Jun N-terminal kinase (JNK) were excluded as relevant CAKs in the context of NF1-depleted ER^+^ breast cancer. Consistent with activation of CDK4/6 by NF1 loss, clinical data indicate that CDK4/6 kinase activities negatively correlate with NF1 protein levels in breast tumors [21].

One common assumption is that higher enzymatic activity confers resistance to drugs targeting that enzyme. Alternatively, elevated activity may reflect an ‘addiction’ essential for survival, thereby creating a therapeutic vulnerability. There are many examples of the latter: HER2^+^ cells are addicted to HER2 signaling, and *BRCA*-mutant cells to poly(ADP-ribose) polymerase (PARP) activity. These vulnerabilities were not initially recognized or exploited; consequently, neither trastuzumab nor olaparib demonstrated significant benefit when first tested without biomarker guidance. The data provided by Zheng et al. suggest that NF1-depleted ER tumors are also addicted to CDK4/6 activity when treated with endocrine therapy (Figure 1). In particular, two NF1-depleted ER^+^ patient-derived xenograft (PDX) models resistant to fulvestrant underwent marked regression when treated with the addition of palbociclib, whereas an NF1-normal model exihibited only cytostatic effects under palbociclib treatment. To validate their findings using a clinical dataset, they examined mRNA levels in biopsy samples from the neoadjuvant NeoPalAna trial (NCT01723774) [26]. In this trial, early-stage ER^+^/HER2^−^ breast cancer patients first received anastrozole for 4 weeks, followed by palbociclib for another two weeks. Biopsies were obtained at baseline, after one month of anastrozole, and after six weeks of combined treatment. Zheng et al. showed that *NF1*-normal ER^+^/HER2^−^ tumors responded to anastrozole as expected, while *NF1*-low tumors were largely resistant to anastrozole alone but regained sensitivity with the anastrozole/palbociclib combination.

A major challenge in assessing the impact of NF1 on treatment response lies in reliably measuring NF1 inactivation in the tumors. While *NF1* mutation analysis is commonly used, it underestimates cases in which NF1 is functionally inactivated. *NF1* mutations are detected in only 2–5% early-stage ER^+^ breast cancers. However, by measuring mRNA expression, as well as protein levels by IHC [27], the frequency of NF1-loss is estimated to be around 25%. Zheng et al. reported another approach to assess *NF1* functional status via copy number loss. Specifically, they report that about 20% of ER^+^/HER2^−^ breast cancers in the METABRIC [28] dataset harbor a shallow *NF1* deletion (−1 copy number) correlating with poorer RFS and disease-specific survival [21].

In solid tumors, a single tumor often carries multiple driver and passenger mutations. To determine whether NF1 inactivation acts alone or cooperates with other oncogenic events in early-stage breast cancer to promote progression to aggressive disease, we analyzed METABRIC data using Fisher’s exact test (Table 1). *NF1* shallow deletions most frequently co-occur with tumor protein 53 (*TP53*) mutations, with an odds ratio of 5.3 (*p* = 1.10 × 10^−58^, Table 1), and both missense and “truncating” *TP53* mutations behave similarly. Co-occurrence of *TP53* mutation and *NF1* loss has also been reported in other cancers, including acute myloid leukemia [29], non-small cell lung cancer [30], and ovarian serous carcinoma [31]. The next most frequent co-occurring events affecting *NF1* itself (odds ratio = 3.3, *p* = 7.50 × 10^−8^, Table 1), suggesting strong selection for NF1 inactivation during progression toward metastasis. Mutations in *USH2A* and *MUC16*, encoding usherin and mucin, rank a distant 3rd and 4th by odds ratio (1.7 and 1.5, respectively, Table 1), but their roles in breast cancer remain unclear. The analysis also reveals mutual exclusivity between *NF1* shallow deletions and mutations in *MAP3K1*, *PIK3CA*, and *GATA3*, which occur more commonly in luminal breast cancers [32]. In primary ER^+^ breast cancer, *PIK3CA* mutations correlate with better outcomes [32], whereas in the metastatic setting they predict poor prognosis [33]; accordingly, the PI3Kα-selective inhibitor alpelisib is FDA-approved for advanced ER^+^ breast cancer [34]. Notably, *NF1* and *PICK3CA* mutations frequently co-occur in a metastatic breast cancer cohort (odds ratio = 1.75, *p* < 0.001) [21], suggesting convergent signaling pathways that drive metastatic progression. Importantly, CDK4/6 remain viable therapeutic targets in tumors resistant to alpelisib, as these tumors continue to respond to CDK4/6i in preclinical models [35].

Notably, co-inactivation of NF1 and p53 is associated with the poorest RFS compared to tumors in which only one genetic defect could be detected (Table 2). Given NF1-p53 co-inactivation is also detected in metastatic breast cancer [21], we speculate that NF1 and p53 inactivation cooperate to plays a key role in promoting metastasis. Co-activation of ER and RAS, driven by NF1 loss, converges on CDK4/6 to promote endocrine resistance in ER^+^ tumors. Concurrent p53 inactivation may further increase CDK4/6 activity by suppressing *CDKN1A* expression (Figure 1), which encodes the CDK4/6 inhibitor p21 (also known as WAF1/CLP1) [36,37,38]. How p53 impacts treatment response to CDK4/6i remains inconclusive, however. Consistent with the concept that p53-inactive tumors require CDK4/6 activity to survive under endocrine therapy, both wild type and mutant *TP53* tumors responded to palbociclib in NeoPalAna [26] and PALOMA-3 [17], as well as ribociclib in MONALEESA-2 [39] trials. In contrast, in an Asian cohort [40], baseline *TP53* mutations correlate with worse outcomes following ribociclib treatment. In addition, genomic instability caused by p53 inactivation may foster additional oncogenic alterations that cooperate with NF1 inactivation or disrupt *NF1* itself, which is a particularly large gene spanning more than 282 kb with 60 exons, making it prone to errors during cell division. In future clinical investigations, NF1 and p53 status may need to be co-analyzed to refine the determination of the subset of patients who could benefit most from CDK4/6i treatment. To this end, we suggest that rather than relying solely on *NF1* mutations, which are rare, we should focus on detecting *NF1* mRNA or protein levels to assess NF1 functional status. For the latter, an IHC assay using a commercially available monoclonal antibody [22] (MABN2557/A376G3) is available and can identify NF1-depleted tumors associated with letrozole resistance [27]. For p53, a combination of IHC and whole exome sequencing may be advisable as missense loss-of-function *TP53* mutations can stabilize p53 protein [41].

CRAF recognizes the specific amino acid sequence in CDK4’s activation loop. This finding raises the possibility that a CDK4-specific inhibitor would be more on-target in driving clinical efficacy in early-stage ER^+^ breast cancer. Data from DepMap, where ER^+^ breast cancer cell lines underwent systematic gene silencing to identify genes essential for survival [42], show that *CDK4* knockout yields much lower DepMap CRISPR CERES scores than *CDK6* knockout [43], indicating stronger dependency on CDK4 than CDK6 for viability. In addition, amplification events are more common with *CDK4* than *CDK6* in primary luminal breast cancers: about 6% of luminal tumors carry *CDK4* amplification, whereas *CDK6* amplification is undetectable [44]. Wild type CRAF can control endocrine therapy by directly activating CDK4, and up to 8% in a cohort of metastatic breast cancers [45] harbor genetic alterations in *CRAF* (e.g., amplification). However, current FDA-approved RAF inhibitors target mutant BRAF, leaving wild type CRAF alterations unaddressed and highlighting an opportunity for future drug development.

In summary, CDK4/6i have fundamentally transformed the therapeutic landscape of ER^+^ breast cancer, yet their broad application is constrained by heterogeneous benefit, cost, and toxicity, underscoring the need for proper patient selection. Functional NF1 loss, present in up to 25% of cases when shallow deletion and reduced mRNA/protein levels are considered, should be assessed both for prognostic significance and for predicting benefit from CDK4/6i in ER^+^ disease. Mechanistically, NF1 loss co-activates ER and RAS signaling, increasing CDK4 activity through cyclin D1 induction and activation-loop phosphorylation. This dual input creates a profound dependency, reframing CDK4/6 activity not as a resistance mechanism but as a therapeutically exploitable vulnerability under endocrine pressure. This framework is supported by clinical data, including observations from the NeoPalAna trial where NF1-low tumors that progressed on anastrozole were re-sensitized upon the addition of palbociclib.

These insights carry clear translational implications. First, biomarker strategies should prioritize robust assays that measure functional NF1 status (e.g., protein levels by IHC or mRNA expression) rather than relying solely on rare mutation analysis, in order to more accurately identify this patient subgroup. We should also develop clinical assays detecting CDK4-pT172 levels. Such assays should be integrated into prospective trials, including neoadjuvant designs with an endocrine lead-in, to evaluate NF1 as a predictive biomarker for risk-stratifying treatment. Second, elucidation of the RAS-CRAF-CDK4-pT172 axis, combined with the greater dependency on CDK4 versus CDK6 in ER^+^ disease, provides a compelling rationale for developing next-generation CDK4-selective inhibitors. In short, the field must pivot from a ‘CDK4/6i for all’ approach to biomarker-anchored, CDK4-centric therapy, with NF1 expression serving as one potential gateway to patient selection and mechanism-informed pharmacology guiding the next wave of precision oncology trials.

## Figures and Tables

**Figure 1:**
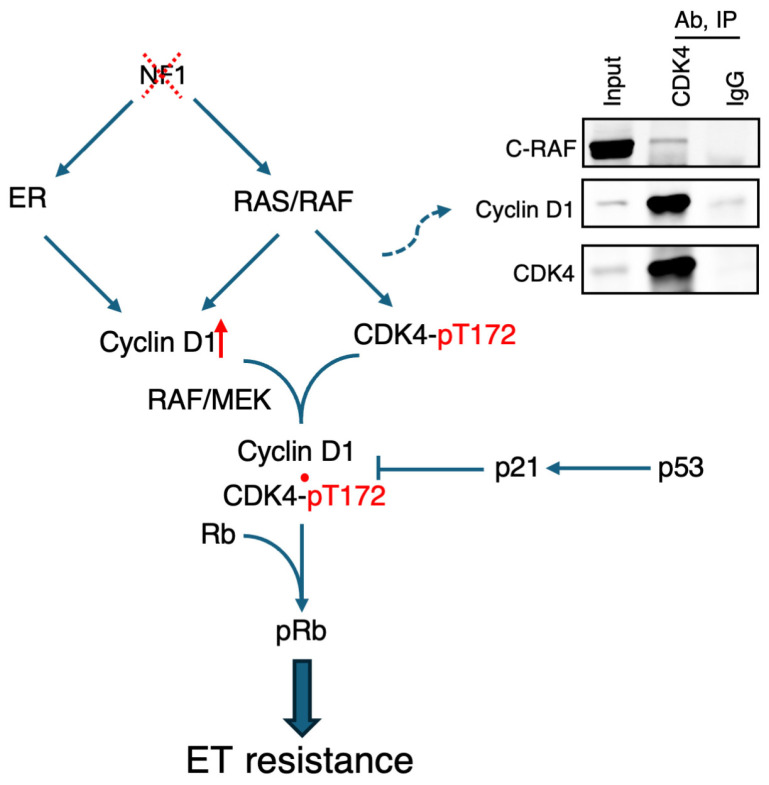
Model. NF1 depletion co-activates ER and RAS signaling, which converge on the CDK4/6-Rb pathway to bypass endocrine therapy by cyclin D1 induction (red upward arrow), and CDK4 phosphorylation at T172. p53 may modulate with this pathway via regulation of p21. **Upper right**: co-immunoprecipitation showed interaction between CDK4 and CRAF in ER^+^ T47D cells.

**Table 1:** Co-occurring and mutually exclusive mutations associated with *NF1* shallow deletion, relative to normal *NF1* copy number, in the METABRIC ER^+^ breast cancer cohort. *p* value and odds ratio were calculated using Fisher’s exact test. FDR was adjusted across all mutations, including non-significant ones. Only mutations with FDR < 0.05 are reported.

Gene	*p* Value	FDR	Odds Ratio	Trend
*TP53*	1.1 × 10^−58^	1.9 × 10^−56^	5.3	Co-occurring
*NF1*	7.5 × 10^−8^	6.5 × 10^−6^	3.2	Co-occurring
*USH2A*	1.3 × 10^−3^	3.3 × 10^−2^	1.7	Co-occurring
*MUC16*	7.3 × 10^−4^	2.1 × 10^−2^	1.5	Co-occurring
*PIK3CA*	4.7 × 10^−4^	1.6 × 10^−2^	0.7	Mutually Exclusive
*GATA3*	1.1 × 10^−4^	4.6 × 10^−3^	0.5	Mutually Exclusive
*MAP3K1*	4.4 × 10^−4^	2.5 × 10^−3^	0.5	Mutually Exclusive

**Table 2:** Relapse-free survival in relation to *NF1* and *TP53* genetic status in the ER^+^ breast cancer cohort in METABRIC. Hazard ratio with 95% confidence intervals (CI), and *p* values were calculated using the Cox proportional-hazards model, with patients having normal *NF1* copy number and wild type *TP53* (*n* = 886) as the reference group.

Tumor Type	Hazard Ratio (HR)	HR 95% CI	*p* Value
*NF1* Loss, *TP53* Wildtype (*n* = 148)	1.64	1.27–2.13	0.000185
*NF1* Normal, *TP53* Mutated (*n* = 127)	1.62	1.22–2.15	0.00077
*NF1* Loss, *TP53* Mutated (*n* = 106)	1.74	1.3–2.35	0.000248
